# Multifactor dimensionality reduction method identifies novel SNP interactions in the WNT protein interaction networks that are associated with recurrence risk in colorectal cancer

**DOI:** 10.3389/fonc.2023.1122229

**Published:** 2023-03-14

**Authors:** Aaron A. Curtis, Yajun Yu, Megan Carey, Patrick Parfrey, Yildiz E. Yilmaz, Sevtap Savas

**Affiliations:** ^1^ Division of Biomedical Sciences, Faculty of Medicine, Memorial University, St. John’s, NL, Canada; ^2^ Institute of Cardiovascular Research, Southwest Medical University, Luzhou, Sichuan, China; ^3^ Discipline of Medicine, Faculty of Medicine, Memorial University, St. John’s, NL, Canada; ^4^ Department of Mathematics and Statistics, Faculty of Science, Memorial University, St. John’s, NL, Canada; ^5^ Discipline of Oncology, Faculty of Medicine, Memorial University, St. John’s, NL, Canada

**Keywords:** Wnt pathway, recurrence, multifactor dimensionality reduction, SNP interactions, colorectal cancer

## Abstract

**Background:**

Interactions among genetic variants are rarely studied but may explain a part of the variability in patient outcomes.

**Objectives:**

In this study, we aimed to identify 1 to 3 way interactions among SNPs from five Wnt protein interaction networks that predict the 5-year recurrence risk in a cohort of stage I-III colorectal cancer patients.

**Methods:**

423 patients recruited to the Newfoundland Familial Colorectal Cancer Registry were included. Five Wnt family member proteins (Wnt1, Wnt2, Wnt5a, Wnt5b, and Wnt11) were selected. The BioGRID database was used to identify the proteins interacting with each of these proteins. Genotypes of the SNPs located in the interaction network genes were retrieved from a genome-wide SNP genotype data previously obtained in the patient cohort. The GMDR 0.9 program was utilized to examine 1-, 2-, and 3-SNP interactions using a 5-fold cross validation step. Top GMDR 0.9 models were assessed by permutation testing and, if significant, prognostic associations were verified by multivariable logistic regression models.

**Results:**

GMDR 0.9 has identified novel 1, 2, and 3-way SNP interactions associated with 5-year recurrence risk in colorectal cancer. Nine of these interactions were multi loci interactions (2-way or 3-way). Identified interaction models were able to distinguish patients based on their 5-year recurrence-free status in multivariable regression models. The significance of interactions was the highest in the 3-SNP models. Several of the identified SNPs were eQTLs, indicating potential biological roles of the genes they were associated with in colorectal cancer recurrence.

**Conclusions:**

We identified novel interacting genetic variants that associate with 5-year recurrence risk in colorectal cancer. A significant portion of the genes identified were previously linked to colorectal cancer pathogenesis or progression. These variants and genes are of interest for future functional and prognostic studies. Our results provide further evidence for the utility of GMDR models in identifying novel prognostic biomarkers and the biological importance of the Wnt pathways in colorectal cancer.

## Background

One of the most common cancers in the world is colorectal cancer (1). This disease includes the cancers of the colon and rectum, has a number of identified genetic and environmental/life-style risk factors, shows geographic difference in incidence and survival rates, and is overall characterized by moderate to low survival rates (1–5). While there are a number of disease and patient related factors that help prognosis (6, 7), identifying additional factors is needed to improve the precision of prognosis (8). Personalized/Precision Medicine approaches can improve prognosis by focusing on new biomarkers. Germline (i.e. not tumor) genetic variations, such as SNPs, are candidate biomarkers, as they are relatively stable, abundant, and show variability among individuals (9). They also have the potential to help identify the biological bases of human conditions and phenotypes. As such, there has been an emphasis on examining SNP–outcome associations in cancers, including in colorectal cancer.

An important aspect of cancer genetics that these analyses miss is that potential interactions among SNPs may be associated with patient outcomes. For example, most of the studies in colorectal cancer have so far focused on individual SNPs’ associations with outcome risk/survival times (10–15). However, it is possible that a SNP’s genotypes may not be associated with the outcome on its own, but they may when they exist together with another SNP’s genotypes (16). Examining interactions can be challenging, however, as the number of variables examined increases, so does the need for computational resources. Methodologies (such as, Multifactor Dimensionality Reduction [MDR]) that address this challenge and tools that utilize these methods (such as GMDR 0.9) have been developed to examine interactions in a relatively feasible way (16–18). We and others have previously shown the utility of MDR and examining interactions among genetic variables in colorectal cancer (19–23). As these studies showed, examining interactions is a promising research area and can reveal new biomarkers that can predict patient outcomes.

Wnt genes have important roles in normal cellular functions as well as colorectal cancer development and its progression (24–26). Therefore, they are excellent biological candidates to examine in colorectal cancer. In this study, our aim was to use a Generalized MDR tool (GMDR 0.9 (18)) to examine the interactions among the germline variables of five Wnt protein interaction networks in relation to 5-year recurrence-free survival status in a cohort of stage I-III colorectal cancer patients.

## Data and methods

### Ethics statement

This study was conducted with ethics approval by the Health Research Ethics Authority of Newfoundland and Labrador (HREB #2018.051; #2009.106). This study was a secondary use of data study, hence, HREB waived the requirement for patient consent.

### Clinical and genetic patient data

The baseline features of the patient cohort are shown in **Table 1**. The patients included in this study were recruited to the Newfoundland Familial Colorectal Cancer Registry (NFCCR) between 1999-2003, and were followed up until 2018 (11, 27, 28). Clinical and pathological data were collected by the NFCCR using medical records, tumor registry data, and other resources as described in other publications (11, 27, 28). Genetic data was a part of the previously obtained genomewide SNP genotype data (29). PLINK (1.7) (30) was used to manage the SNP genotype data used in this study. The 5-year local or distant recurrence-free survival (RMFS) status was the response variable. All patients were unrelated to each other and of Caucasian background (29).

**Table 1:** Baseline characteristics of the patient cohort included in the GMDR and logistic regression analysis.

Variable	N (Total 423)	%
Tumor Location		
Colon	270	64
Rectum	153	36
Stage		
I	81	19
II	185	44
III	157	37
Adjuvant Chemotherapy		
Yes	250	59
No	173	41
Adjuvant Radiotherapy		
Yes	116	27
No	307	73
5-Year RMFS Status		
Local or distant recurrence (-)	315	74
Local or distant recurrence (+)	108	26

RMFS, local or distant recurrence free survival.

### Identification of Wnt interactome networks using the BioGRID database

We focused on five WNT genes in non-canonical/β-catenin independent pathway: *Wnt1*, *Wnt2*, *Wnt5a*, *Wnt5b*, and *Wnt11* (25). We utilized the BioGRID database (31) to retrieve the interaction networks for each of these genes. **Table S1** shows the genes in each protein interaction network after implementing quality control measures [described in detail in Curtis et al. (23)]. We then used the SNP genotype data and PLINK to retrieve the SNPs located in these genes using the following criteria: Minor Allele Frequency (MAF) >= 0.05; Hardy-Weinberg Equilibrium (HWE) > 0.0001; and missing genotype data = 0%. Pruning was performed by PLINK to remove the SNPs in high-LD, as explained in Curtis et al. (23). As a result we ended up with 6 (in *Wnt1*), 18 (in *Wnt2*), 53 (in *Wnt5a*), 4 (in *Wnt5b*), and 20 (in *Wnt11*) genes respectively (**Supplementary Table S1** **)**, and around 1,000 SNPs in these Wnt interaction networks (**Supplementary Table S2**).

### GMDR 0.9 runs, permutation testing, and statistical analyses

**Figure 1:**
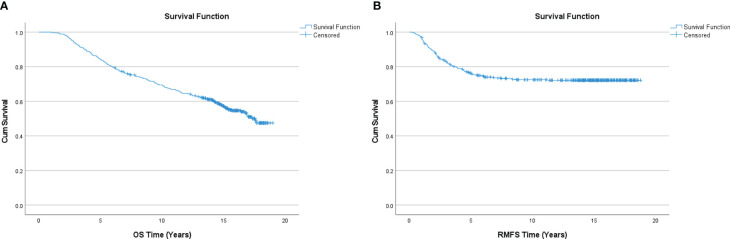
**(A)** Kaplan Meier curve for overall survival (OS). **(B)** Kaplan Meier curve for local or distant recurrence-free survival (RMFS).

GMDR 0.9 (18, 32) was previously downloaded from the UAB Department of Biostatistics Section on Statistical Genetics website. All analyses were done as described in Curtis et al. (23). In brief, we have conducted 1-way interactions (examining the interactions among the three potential genotypes of single SNPs); 2-way interactions (examining the interactions among the genotypes of two SNPs); and 3-way interactions (examining the interactions among the genotypes of three SNPs). Known prognostic markers, disease stage, tumor location, and adjuvant chemotherapy and radiotherapy statuses were used as variables. A 5-step cross-validation was implemented; where the dataset was partitioned into five parts, with four parts used as the training cohort and the remaining 5^th^ part used as the testing (i.e. validation) cohort. Each of the five partitions was designated the testing cohort once and the results for each of these datasets were compared. GMDR analysis was repeated 20 times, using different random seeds, and the MDR model that was identified most frequently and with the highest Testing Balance Accuracy (TBA) score was selected as the top model for the examined interaction. Rarely, we used the higher Cross Validation Consistency (CVC) and specificity data to break a tie. We performed 1-way analysis iteratively for each dataset, and if a model was found to be significant (i.e. a SNP with a main effect was identified in the dataset), such SNPs were removed from the dataset. This was repeated until no significant 1-way model was found (16, 33). The significance of the top model was then examined using permutation testing. Models that were significant (p < 0.001) were examined in multivariable logistic regression models, adjusting for disease stage, tumor location, and adjuvant chemotherapy and radiotherapy status. A p-value < 0.05 was considered significant in multivariable logistic regression models. Kaplan Meier curves were created for overall survival (OS) and RMFS using the long-term follow up data (28) and end point status (in OS, the end point was death from any cause). In addition, 18 patients who were censored before or at the 5 year time point and as such were excluded from the GMDR and logistic regression analyses, were included in the Kaplan Meier analyses to limit bias. **Figure 1** shows the OS and RMFS curves for these patients. The 5-year OS probability was around 80% and a little bit less than that for the RMFS. R (version 3.5.3) (34) and SPSS (version 28.0.0.0 (190); 35) were used for data processing and statistical analyses.

### Functional annotation of genes and SNPs using databases

Identified SNPs and genes were examined for their functional and biological features using literature and databases. Specifically, SNPs that were identified in 1-SNP, 2-SNP, and 3-SNP interaction analyses were checked for their functional consequences in RegulomeDB (v2.0.3) (36), which uses a ranking system to describe the regulatory potential of SNPs (ranks 1a-1f specify expression quantitative trait loci [eQTLs]). In addition, SNPs were searched in the GTEx (data release v8) (37) database to see whether they were eQTLs. The latter database provided eQTL data for colon tissues only. The dbCPCO database (38) was utilized to search whether the identified SNPs were previously reported to be associated with clinical outcomes in colorectal cancer. Information about the genes/proteins was collected from literature and the Gene Entrez database (39). The dbSNP database (40) was utilized to retrieve information on SNP annotations (e.g. intronic, missense).

## Results

The clinical features of the patient cohort are summarized in **Table 1**.

By examining around 26,298,702 interactions (2-SNP interactions=173,425; 3-SNP interactions=26,125,277) in five Wnt interactome networks, our investigation identified 32 novel interactions associated with 5-year RMFS status in colorectal cancer.

**Figure 2:**
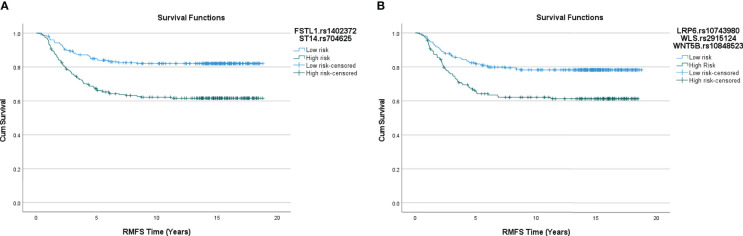
**(A)** Kaplan Meier curve for the Wnt5a interactome, 2-way interaction. Log rank p-value: 2.143x10^-6^. **(B)** Kaplan Meier curve for the Wnt5a interactome, 3-way interaction. Log rank p-value: 1.296 x10^-4^.

Interactions were identified in each of the networks examined. Twenty-three 1-SNP interactions, where the genotypes of individual SNPs were identified as associated with the 5-year RMFS status in multivariable logistic regression models are shown in **Supplementary Table S3**. Additionally, we identified nine multi-SNP interactions (four 2-way and five 3-way interactions) that were associated with the 5-year RMFS status in the patient cohort, when adjusted for other prognostic markers in the logistic regression models (**Table 2**). Kaplan Meier curves for these interactions are shown in **Figure 2** (for the Wnt5a network) and **Supplementary Figure S1** (for the Wnt1, Wnt2, Wnt5b, and Wnt11 networks).

Table 2Results of the two-way and three-way interaction analyses.

**Table:** 

a) Wnt1										
µTop Model SNPs	Top Model Risk Categorization	Permutation Testing P-value		Logistic Regression P-value				Odds Ratio (OR)	Logistic Regression 95% Confidence Interval (CI)	
2-way										
ROR2.rs7037255_A, SFRP1.rs7843510_G	rs7037255_A = GG and rs7843510_G = GA or rs7037255_A = AG and rs7843510_G = GG or rs7037255_A = AA and rs7843510_G = AA or rs7037255_A = AA and rs7843510_G = GA or rs7037255_A = AA and rs7843510_G = GG → Low Risk rs7037255_A = GG and rs7843510_G = AA or rs7037255_A = GG and rs7843510_G = GG or rs7037255_A = AG and rs7843510_G = AA or rs7037255_A = AG and rs7843510_G = GA → High Risk	<0.001		4.587E-06				3.362	2.002 - 5.647	
3-way										
LRP6.rs11609634_T, ROR2.rs7037255_A, UBR3.rs11691281_G	rs11609634_T = CC and rs7037255_A = GG and rs11691281_G = TT or rs11609634_T = CC and rs7037255_A = GG and rs11691281_G = GT or rs11609634_T = CC and rs7037255_A = AG and rs11691281_G = GG or rs11609634_T = CC and rs7037255_A = AA and rs11691281_G = TT or rs11609634_T = CC and rs7037255_A = AA and rs11691281_G = GT or rs11609634_T = CC and rs7037255_A = AA and rs11691281_G = GG or rs11609634_T = TC and rs7037255_A = GG and rs11691281_G = TT or rs11609634_T = TC and rs7037255_A = AA and rs11691281_G = TT or rs11609634_T = TC and rs7037255_A = AA and rs11691281_G = GT or rs11609634_T = TC and rs7037255_A = AA and rs11691281_G = GG or rs11609634_T = TT and rs7037255_A = GG and rs11691281_G = TT or rs11609634_T = TT and rs7037255_A = GG and rs11691281_G = GT or rs11609634_T = TT and rs7037255_A = GG and rs11691281_G = GG or rs11609634_T = TT and rs7037255_A = AG and rs11691281_G = TT or rs11609634_T = TT and rs7037255_A = AG and rs11691281_G = GG or rs11609634_T = TT and rs7037255_A = AA and rs11691281_G = TT or rs11609634_T = TT and rs7037255_A = AA and rs11691281_G = GG → Low Risk rs11609634_T = CC and rs7037255_A = GG and rs11691281_G = GG or rs11609634_T = CC and rs7037255_A = AG and rs11691281_G = TT or rs11609634_T = CC and rs7037255_A = AG and rs11691281_G = GT or rs11609634_T = TC and rs7037255_A = GG and rs11691281_G = GT or rs11609634_T = TC and rs7037255_A = GG and rs11691281_G = GG or rs11609634_T = TC and rs7037255_A = AG and rs11691281_G = TT or rs11609634_T = TC and rs7037255_A = AG and rs11691281_G = GT or rs11609634_T = TC and rs7037255_A = AG and rs11691281_G = GG or rs11609634_T = TT and rs7037255_A = AG and rs11691281_G = GT or rs11609634_T = TT and rs7037255_A = AA and rs11691281_G = GT → High Risk	<0.001		9.407E-09				4.573	2.722 - 7.685

**Table:** 

b) Wnt2										
µTop Model SNPs	Top Model Risk Categorization		Permutation Testing P-value		Logistic Regression P-value			Odds Ratio (OR)		Logistic Regression Confidence Interval (CI)
2-way										
GPC1.rs12695020_G, WLS.rs2116046_C	rs12695020_G = AA and rs2116046_C = TT or rs12695020_G = AA and rs2116046_C = CC or rs12695020_G = GA and rs2116046_C = CT or rs12695020_G = GA and rs2116046_C = CC or rs12695020_G = GG and rs2116046_C = TT → Low Risk rs12695020_G = AA and rs2116046_C = CT or rs12695020_G = GA and rs2116046_C = TT or rs12695020_G = GG and rs2116046_C = CT or rs12695020_G = GG and rs2116046_C = CC → High Risk		0.004		0.00004949			2.640		1.652 - 4.220
3-way										
HCK.rs980368_G, PPP6R3.rs2840367_C, SORL1.rs3862606_G	rs980368_G = AA and rs2840367_C = CT and rs3862606_G = AA or rs980368_G = AA and rs2840367_C = CT and rs3862606_G = GA or rs980368_G = AA and rs2840367_C = CC and rs3862606_G = AA or rs980368_G = AA and rs2840367_C = CC and rs3862606_G = GA or rs980368_G = GA and rs2840367_C = TT and rs3862606_G = AA or rs980368_G = GA and rs2840367_C = CT and rs3862606_G = GA or rs980368_G = GA and rs2840367_C = CT and rs3862606_G = GG or rs980368_G = GA and rs2840367_C = CC and rs3862606_G = GG or rs980368_G = GG and rs2840367_C = TT and rs3862606_G = GA or rs980368_G = GG and rs2840367_C = TT and rs3862606_G = GG or rs980368_G = GG and rs2840367_C = CT and rs3862606_G = AA or rs980368_G = GG and rs2840367_C = CT and rs3862606_G = GA or rs980368_G = GG and rs2840367_C = CC and rs3862606_G = AA or rs980368_G = GG and rs2840367_C = CC and rs3862606_G = GA → Low Risk rs980368_G = AA and rs2840367_C = TT and rs3862606_G = AA or rs980368_G = AA and rs2840367_C = TT and rs3862606_G = GA or rs980368_G = AA and rs2840367_C = TT and rs3862606_G = GG or rs980368_G = AA and rs2840367_C = CT and rs3862606_G = GG or rs980368_G = AA and rs2840367_C = CC and rs3862606_G = GG or rs980368_G = GA and rs2840367_C = TT and rs3862606_G = GA or rs980368_G = GA and rs2840367_C = TT and rs3862606_G = GG or rs980368_G = GA and rs2840367_C = CT and rs3862606_G = AA or rs980368_G = GA and rs2840367_C = CC and rs3862606_G = AA or rs980368_G = GA and rs2840367_C = CC and rs3862606_G = GA or rs980368_G = GG and rs2840367_C = TT and rs3862606_G = AA or rs980368_G = GG and rs2840367_C = CT and rs3862606_G = GG → High Risk		<0.001		9.244E-10			4.933		2.959 - 8.222

**Table:** 

c) Wnt5a										
µTop Model SNPs	Top Model Risk Categorization		Permutation Testing P-value		Logistic Regression P-value			Odds Ratio (OR)		Logistic Regression Confidence Interval (CI)
2-way										
FSTL1.rs1402372_T, ST14.rs704625_G	rs1402372_T = GG and rs704625_G = GG or rs1402372_T = TG and rs704625_G = GC or rs1402372_T = TG and rs704625_G = GG or rs1402372_T = TT and rs704625_G = CC or rs1402372_T = TT and rs704625_G = GG → Low Risk rs1402372_T = GG and rs704625_G = CC or rs1402372_T = GG and rs704625_G = GC or rs1402372_T = TG and rs704625_G = CC or rs1402372_T = TT and rs704625_G = GC → High Risk		<0.001			1.270E-06		3.281		2.029 - 5.306
3-way										
LRP6.rs10743980_T, WLS.rs2915124_C, WNT5B.rs10848523_A	rs10743980_T = CC and rs2915124_C = TT and rs10848523_A = AG or rs10743980_T = CC and rs2915124_C = TT and rs10848523_A = AA or rs10743980_T = CC and rs2915124_C = CT and rs10848523_A = GG or rs10743980_T = CC and rs2915124_C = CT and rs10848523_A = AG or rs10743980_T = CC and rs2915124_C = CC and rs10848523_A = AA or rs10743980_T = TC and rs2915124_C = TT and rs10848523_A = AG or rs10743980_T = TC and rs2915124_C = TT and rs10848523_A = AA or rs10743980_T = TC and rs2915124_C = CT and rs10848523_A = GG or rs10743980_T = TC and rs2915124_C = CT and rs10848523_A = AA or rs10743980_T = TC and rs2915124_C = CC and rs10848523_A = GG or rs10743980_T = TC and rs2915124_C = CC and rs10848523_A = AG or rs10743980_T = TC and rs2915124_C = CC and rs10848523_A = AA or rs10743980_T = TT and rs2915124_C = TT and rs10848523_A = GG or rs10743980_T = TT and rs2915124_C = CT and rs10848523_A = GG or rs10743980_T = TT and rs2915124_C = CT and rs10848523_A = AG or rs10743980_T = TT and rs2915124_C = CC and rs10848523_A = AG → Low Risk rs10743980_T = CC and rs2915124_C = TT and rs10848523_A = GG or rs10743980_T = CC and rs2915124_C = CT and rs10848523_A = AA or rs10743980_T = CC and rs2915124_C = CC and rs10848523_A = GG or rs10743980_T = CC and rs2915124_C = CC and rs10848523_A = AG or rs10743980_T = TC and rs2915124_C = TT and rs10848523_A = GG or rs10743980_T = TC and rs2915124_C = CT and rs10848523_A = AG or rs10743980_T = TT and rs2915124_C = TT and rs10848523_A = AG or rs10743980_T = TT and rs2915124_C = TT and rs10848523_A = AA or rs10743980_T = TT and rs2915124_C = CT and rs10848523_A = AA or rs10743980_T = TT and rs2915124_C = CC and rs10848523_A = GG → High Risk		<0.001			1.755E-12		6.193		3.731 - 10.28

**Table:** 

d) Wnt5b										
µTop Model SNPs	Top Model Risk Categorization		Permutation Testing P-value			Logistic Regression P-value		Odds Ratio (OR)		Logistic Regression Confidence Interval (CI)
2-way										
WNT5B.rs10773958_A, WNT5B.rs10491958_T	rs10773958_A = GG and rs10491958_T = CC or rs10773958_A = AG and rs10491958_T = TC or rs10773958_A = AG and rs10491958_T = TT or rs10773958_A = AA and rs10491958_T = CC → Low Risk rs10773958_A = GG and rs10491958_T = TC or rs10773958_A = GG and rs10491958_T = TT or rs10773958_A = AG and rs10491958_T = CC or rs10773958_A = AA and rs10491958_T = TC or rs10773958_A = AA and rs10491958_T = TT → High Risk		0.084			–		–		–
3-way										
KLRG2.rs9632774_A, WNT5B.rs11061856_T, WNT5B.rs4766399_G	rs9632774_A = GG and rs11061856_T = CC and rs4766399_G = GT or rs9632774_A = GG and rs11061856_T = TC and rs4766399_G = TT or rs9632774_A = GG and rs11061856_T = TC and rs4766399_G = GG or rs9632774_A = GG and rs11061856_T = TT and rs4766399_G = GT or rs9632774_A = AG and rs11061856_T = CC and rs4766399_G = TT or rs9632774_A = AG and rs11061856_T = TC and rs4766399_G = GT or rs9632774_A = AG and rs11061856_T = TC and rs4766399_G = GG or rs9632774_A = AA and rs11061856_T = CC and rs4766399_G = TT or rs9632774_A = AA and rs11061856_T = CC and rs4766399_G = GG or rs9632774_A = AA and rs11061856_T = TC and rs4766399_G = GT or rs9632774_A = AA and rs11061856_T = TC and rs4766399_G = GG or rs9632774_A = AA and rs11061856_T = TT and rs4766399_G = GT → Low Risk rs9632774_A = GG and rs11061856_T = CC and rs4766399_G = TT or rs9632774_A = GG and rs11061856_T = CC and rs4766399_G = GG or rs9632774_A = GG and rs11061856_T = TC and rs4766399_G = GT or rs9632774_A = AG and rs11061856_T = CC and rs4766399_G = GT or rs9632774_A = AG and rs11061856_T = CC and rs4766399_G = GG or rs9632774_A = AG and rs11061856_T = TC and rs4766399_G = TT or rs9632774_A = AA and rs11061856_T = CC and rs4766399_G = GT or rs9632774_A = AA and rs11061856_T = TC and rs4766399_G = TT → High Risk		0.011			2.894E-07		3.561		2.192 - 5.786

**Table:** 

e) Wnt11										
µTop Model SNPs	Top Model Risk Categorization		Permutation Testing P-value				Logistic Regression P-value	Odds Ratio (OR)		Logistic Regression Confidence Interval (CI)
2-way										
FUCA2.rs11155297_T, TMED7.rs10075869_G	rs11155297_T = GG and rs10075869_G = GA or rs11155297_T = TG and rs10075869_G = AA → Low Risk rs11155297_T = GG and rs10075869_G = AA or rs11155297_T = GG and rs10075869_G = GG or rs11155297_T = TG and rs10075869_G = GA or rs11155297_T = TG and rs10075869_G = GG or rs11155297_T = TT and rs10075869_G = AA or rs11155297_T = TT and rs10075869_G = GA or rs11155297_T = TT and rs10075869_G = GG → High Risk		0.004				5.879E-06	3.328		1.978 - 5.598
3-way										
C1orf54.rs10157197_A, TMED7.rs698366_A, WNT11.rs17749202_C	rs10157197_A = GG and rs698366_A = CC and rs17749202_C = TT or rs10157197_A = GG and rs698366_A = CC and rs17749202_C = CT or rs10157197_A = GG and rs698366_A = AC and rs17749202_C = CT or rs10157197_A = GG and rs698366_A = AC and rs17749202_C = CC or rs10157197_A = GG and rs698366_A = AA and rs17749202_C = TT or rs10157197_A = GG and rs698366_A = AA and rs17749202_C = CC or rs10157197_A = AG and rs698366_A = CC and rs17749202_C = CC or rs10157197_A = AG and rs698366_A = AC and rs17749202_C = TT or rs10157197_A = AG and rs698366_A = AC and rs17749202_C = CT or rs10157197_A = AG and rs698366_A = AC and rs17749202_C = CC or rs10157197_A = AG and rs698366_A = AA and rs17749202_C = TT or rs10157197_A = AA and rs698366_A = CC and rs17749202_C = CT or rs10157197_A = AA and rs698366_A = CC and rs17749202_C = CC or rs10157197_A = AA and rs698366_A = AC and rs17749202_C = TT or rs10157197_A = AA and rs698366_A = AC and rs17749202_C = CC or rs10157197_A = AA and rs698366_A = AA and rs17749202_C = CT → Low Risk rs10157197_A = GG and rs698366_A = CC and rs17749202_C = CC or rs10157197_A = GG and rs698366_A = AC and rs17749202_C = TT or rs10157197_A = GG and rs698366_A = AA and rs17749202_C = CT or rs10157197_A = AG and rs698366_A = CC and rs17749202_C = TT or rs10157197_A = AG and rs698366_A = CC and rs17749202_C = CT or rs10157197_A = AG and rs698366_A = AA and rs17749202_C = CT or rs10157197_A = AG and rs698366_A = AA and rs17749202_C = CC or rs10157197_A = AA and rs698366_A = CC and rs17749202_C = TT or rs10157197_A = AA and rs698366_A = AC and rs17749202_C = CT or rs10157197_A = AA and rs698366_A = AA and rs17749202_C = TT or rs10157197_A = AA and rs698366_A = AA and rs17749202_C = CC → High Risk		0.001				5.655E-09	4.149		2.571 - 6.695

High risk genotypes are shown in red font. µThe letter at the end is the minor allele. - says that there is no results to report.

In all logistic regression models, the direction of the Odds Ratios (ORs) were consistent with the high-risk and low-risk genotype combinations identified in the top MDR models.

As observed in other studies, as the order of interactions increased (from 1-SNP to 3-SNP), the significance of the association detected increased as well. In other words, the 3-SNP interaction models were the ones that best separated patients based on their 5-year RMFS status (p-values 10^-7^ to 10^-12^). ORs were also higher in 3-way models compared 1-SNP and 2-SNP models (**Table 2**).

All SNPs identified in interaction models were common in the patient cohort (MAF ≥ 5%). Only one of the SNPs (FUCA2. rs11155297, NP_114409.2:p.Ala233Glu) was a missense variant, whereas the other variants were intronic/non-coding or 3’-UTR variants (**Supplementary Table S4**). Interestingly, some of the SNPs were predicted to be functional (i.e. eQTLs) based on the RegulomeDB scores or GTEx data (**Table 3**). Another interesting finding was that both of the SNPs in a 2-way interaction were eQTLs (in sigmoid and transverse colon tissues): FUCA2.rs11155297 (eQTL for *ADAT2*) and TMED7.rs10075869 (eQTL for *AC010226.4*). Some of the genes in the interaction networks as well as those that are associated with eQTLs had literature findings indicative of their involvement in pathogenesis of colorectal cancer or its progression (**Supplementary Table S5**).

**Table 3:** eQTL information for the SNPs identified.

µVariant	*Chr	*Location (hg19)	**RegulomeDB rank	***GTEx eQTL in colon sigmoid - target gene (chr)	***GTEx eQTL in colon transverse - target gene (chr)
1-WAY					
HSPA5.rs12009_C	9	127997302-127997303	**#1b**	PRPS1P2 (chr9)	PRPS1P2 (chr9)
MKRN2.rs5746255_C	3	12624320-12624321	**#1f**	MKRN2 (chr3)	MKRN2 (chr3)
DDX58.rs944582_G	9	32465769-32465770	7	GVQW1 (chr9); ACO1 (chr9)	DDX58 (chr9); ACO1 (chr9)
DDX58.rs4384073_G	9	32474689-32474690	5	ACO1 (chr9)	No
NME7.rs1080266_A	1	169176589-169176590	5	NME7 (chr1)	NME7 (chr1)
2-WAY					
ROR2.rs7037255_A	9	94696953-94696954	5	No	SPTLC1 (chr9)
SFRP1.rs7843510_G	8	41135768-41135769	5	No	No
GPC1.rs12695020_G	2	241403956-241403957	3a	n/a	n/a
WLS.rs2116046_C	1	68662251-68662252	7	No	****GNG12-AS1 (chr1)
FUCA2.rs11155297_T	6	143825103-143825104	5	ADAT2 (chr6)	ADAT2 (chr6)
TMED7.rs10075869_G	5	114956958-114956959	5	AC010226.4 (chr5)	AC010226.4 (chr5)
3-WAY					
LRP6.rs11609634 _T	12	12309686-12309687	4	No	No
ROR2.rs7037255_A	9	94696953-94696954	5	No	SPTLC1 (chr9)
UBR3.rs11691281_G	2	170861133-170861134	7	No	No
HCK.rs980368_G	20	30681542-30681543	4	No	RP11-358N2.2 (chr20)
PPP6R3.rs2840367_C	11	68302100-68302101	7	No	No
SORL1.rs3862606_G	11	121330086-121330087	3a	No	No
C1orf54.rs10157197_A	1	150250635-150250636	3a	MRPS21 (chr1)	MRPS21 (chr1)
TMED7.rs698366_A	5	114951139-114951140	7	AC010226.4 (chr5); TICAM2 (chr5)	AC010226.4 (chr5); TICAM2 (chr5)
WNT11.rs17749202_C	11	75897373-75897374	2b	No	No

*Based on the RegulomeDB database (36) **The RegulomeDB (36) rank scores signifying eQTLs are shown in bold. ***Based on the GTEx database (data release v8) (37). ****According to the dbSNP database (40), this SNP is also located in GNG12-AS1. #According to RegulomeDB, two of these SNPs (HSPA5.rs12009_C and MKRN2.rs5746255_C) are eQTLs for RABEPK (Rab9 effector protein with kelch motifs), and PPARG (peroxisome proliferator activated receptor gamma) and MKRN2 (makorin ring finger protein 2), respectively, in monocytes. Therefore, in this table and manuscript, we prioritize and focus on the data retrieved from the GTEx database where the tissues are colon. n/a: variant was not found in the GTEx database. µThe letter at the end is the minor allele.

## Discussion

As a globally common disease with moderate to low survival rates (1–5), it is critical to identify new biomarkers that can help prognosis in colorectal cancer. One under-studied but promising research area is that of interactions, where multiple variables together relate to a phenotype, such as recurrence status in cancer patients. Here, we report our study that used GMDR 0.9 (18), a data reduction tool, to examine interactions among different SNPs from the Wnt interactome genes in a cohort of colorectal cancer patients from Newfoundland and Labrador (27). As a result, we were able to identify novel SNP interactions in the five WNT interactome sets that were predictive of 5-year RMFS status in colorectal cancer.

Our results underline the utility of MDR in identifying previously unknown interactions and potential prognostic biomarkers. As shown in **Table 2** and **Supplementary Table S3**, GMDR 0.9, permutation testing, and multivariable logistic regression analyses identified interactions that can distinguish patients based on their 5-year RMFS risk when adjusted for prognostic covariates. Note that high and low risk patient group classifications made by GMDR 0.9 were supported by the direction of effect (i.e. ORs) in the regression models. This increases the confidence in the GMDR 0.9 risk classification system. Among the interactions identified, 2-SNP and 3-SNP interactions (n=9) are the most interesting ones, as they significantly contribute knowledge to the largely unknown multi-loci interactions in colorectal cancer. According to the dbCPCO database (38), only one of the SNPs identified in this study – HSPA5.rs12009 – was investigated in relation to colorectal cancer outcomes in a stage II-III patient cohort. It was not associated with either stage or time-to-recurrence in the study patient cohort which consisted of mixed-ethnicities (41). Of note, in our study HSPA5.rs12009 was identified in both WNT2 and WNT5A pathway analyses and is an eQTL. Prognostic associations of this and other SNPs identified in this study can be verified in additional colorectal cancer cohorts.

It is hard to predict the biological reasons behind such interactions without detailed experimental analyses. However, whether or not these SNPs are likely to have biological roles in the phenotype can be first assessed using existing information, for example, based on their genomic/genic locations, predictive tools/databases, and previously conducted large-scale experiments (such as eQTL analyses). In this regard, RegulomeDB (36) and GTEx (37) database information suggested that a number of the identified variants were eQTLs that were associated with the expression levels of genes. In four cases, these genes included the genes that the SNPs were located in. For example, WLS.rs2116046 was an eQTL for *GNG12-AS1* as well as *WLS* in transverse colon (note that sequences of *WLS* and *GNG12-AS1* overlap, and rs2116046 is also located in *GNG12-AS1*) (**Table 3**). An interesting case was identified in the 2-way interaction results where both of the SNPs identified turned out to be eQTLs: FUCA2.rs11155297 (eQTL for *ADAT2* in both sigmoid and transverse colon) and TMED7.rs10075869 (eQTL for *AC010226.4* in both sigmoid and transverse colon). Little is known about the latter gene but *ADAT2* is involved in tRNA modification (42), upregulated through translational control by BRCA1 and a marker of BRCA1 depletion in cell lines and human breast tumors (43), identified as over-expressed in different solid tumors (44), and linked to malignant transformation through its roles in affecting chromatin, transcriptional processes, and apoptosis (44). Also, while neither *TMED7* (transmembrane p24 trafficking protein 7) nor *FUCA2* (alpha-L-fucosidase 2) genes are known to be linked to colorectal cancer, FUCA2 is associated with various cancers, tumor microenvironment and prognostic features (45). Although this is the first time ADAT2 has been linked to colorectal cancer, our data and literature information make ADAT2 (as well as FUCA2.rs11155297) interesting candidates for future studies in colorectal cancer progression and prognosis.

Additional genes are worth discussion. **Supplementary Table S5** shows the information collected about the genes in the five Wnt interactome networks as well as the genes associated with the eQTL SNPs. A significant portion of the genes was already linked to colorectal cancer pathogenesis or progression/prognosis by previous studies. For example, expression levels, deletion, or biological functions of ROR2, SFRP1, LRP6, PITX2, WLS, GPC1, HCK, HPN, MKRN2, and DDX58 were associated with disease features, patient outcomes/prognosis, or invasive and other malignant features of colorectal tumors (46–57). This literature information supports our findings and can be partly attributed to the fact that the Wnt pathway is one of the most studied pathways in colorectal cancer, increasing the chances of finding literature information on genes functioning in Wnt-related biological processes. Overall, future biological studies and/or interventions can be planned for the genes and eQTLs identified by our analyses.

This study has a number of strengths and limitations. This is one of the few large-scale studies examining such a large number of interactions in colorectal cancer. Interactions identified are novel. The patient cohort is a well annotated cohort and the genes selected have been previously shown to have abnormalities/functional roles in colorectal cancer development and or progression (24–26). The 5-step cross validation and repeating the analyses 20 times helped reduce the false-positive findings, in addition to the permutation testing. Additionally, we examined SNP interactions in protein interaction networks, increasing the biological plausibility of the identified interactions. Our cohort, however, consists of only Caucasian patients, therefore, our results may not be applicable to other ethnicities/populations. The identified variants associated with the 5-year recurrence-free survival need to be verified in other patient cohorts for generalizability of our findings. The study focused on common (MAFs >=5%) SNPs from the autosomal chromosomes, and hence, missed examining the associations of rare variables and variables from sex-chromosomes. As we reported earlier, GMDR 0.9 has certain limitations, so it may have missed interactions (23). However, use of permutation testing, repeating the MDR procedure and choosing the most frequently identified MDR model for each examined interaction, and multivariable regression modeling also have limited the false-positive findings.

In conclusion, we present novel 1 to 3 way SNP interactions that predict the 5-year RMFS status in colorectal cancer. These interactions are excellent candidates for further verification in other patient cohorts. We also identified a number of genes that are biologically linked to colorectal cancer: they form an exciting set for future studies or interventions in colorectal cancer. Our results also indicate that MDR and other data reduction methods should be utilized more widely for comprehensive investigations of statistical interactions in patient prognosis. Finally, our findings also re-emphasize and strengthen the importance of Wnt protein pathways in colorectal cancer.

## Data availability statement

Data that support the findings of this study are available from the Newfoundland Colorectal Cancer Registry/Memorial University of Newfoundland. However, restrictions apply to the availability of this data, and so data are not publicly available. The data used in this study cannot be made publicly available as patients were not consented to make their data publicly available or accessible. Clinical and genetic data are available from the Newfoundland Colorectal Cancer Registry (NFCCR) upon reasonable request for researchers who meet the criteria for access to confidential data. Permission to obtain the data can be requested from Newfoundland Colorectal Cancer Registry (PP; pparfrey@mun.ca) and Research, Grant, and Contract Services (rgcs@mun.ca) at Memorial University of Newfoundland, St. John’s, NL, Canada, and the ethics approval shall be obtained from the Health Research Ethics Board (HREB), Ethics Office, Health Research Ethics Authority, Suite 200, 95 Bonaventure Avenue, St. John’s, NL, A1B 2X5, Canada. The GMDR 0.9 code can be requested from the developers, Drs. Xiang-Yang Lou, Jun Zhu, or Ming D. Li. Requests to access these datasets should be directed to PP; pparfrey@mun.ca) and Research, Grant, and Contract Services (rgcs@mun.ca).

## Ethics statement

The studies involving human participants were reviewed and approved by Health Research Ethics Authority of Newfoundland and Labrador. Written informed consent for participation was not required for this study in accordance with the national legislation and the institutional requirements.

## Author contributions

AC: performed all MDR and statistical analyses; managed the data; helped interpret the results and draft the manuscript: YY: performed the bioinformatics analyses; helped interpret the results and draft the manuscript; MC: helped collect the outcome data; PP: led the NFCCR and helped collect the clinical and genetic patient data: YEY: helped with statistical approach and permutation testing. SS: conceived the idea; supervised the assistants; helped interpret the results; drafted; finalized and submitted the manuscript. All authors contributed to the article and approved the submitted version.

## Supplementary material

Supplementary Table 1 Proteins in the WNT interaction networks.

Supplementary Table 2 SNPs included in this study.

Supplementary Table 3 Single SNP interaction models identified and results of the logistic regression analyses.

Supplementary Table 4 SNP functional annotations (based on the dbSNP database)

Supplementary Table 5 Information on the genes.

Supplementary Figure 1 Kaplan Meier curves for select 2-way and 3-way interactions.
