# Supplementary material for: Multifactor dimensionality reduction method identifies novel SNP interactions in the WNT protein interaction networks that are associated with recurrence risk in colorectal cancer
Source: Front Oncol. 2023 Mar 14;13:1122229. doi: 10.3389/fonc.2023.1122229 (PMC10043327; doi:10.3389/fonc.2023.1122229)
Supplement: Supplementary Table 1 — Proteins in the WNT interaction networks. [file DataSheet_1.pdf]

Table S1. Proteins in the WNT interaction networks.

Interactome	Genes
WNT1	*LRP6 POLR2C ROR2 SFRP1 UBR3 WIF1*
WNT2	*AQP5 DCAF4 GPC1 HCK HSPA5 NME7 NOTCH1 NOTCH2 PASK PITX2 PPP6R2 PPP6R3 RRAGC SFRP1 SORL1 WLS WNT2 ZZEF1*
WNT5A	*ANTXR1 ASAH1 C1QA C7orf34 CD1A CHRNB2 CLEC12B CNNM4 COL6A1 EPHA7 FSTL1 FZD1 GAS6 GGT7 HPN HSPA5 HSPD1 KLRG2 LCN6 LDLRAD1 LGALS3BP LMF1 LRP6 LY6H LY86 LYPD1 MKRN2 MSMB MYCBP2 NOTCH2 NPTX1 PDGFRA PITX2 PPT1 PROC PROS1 PSCA PTPRK SCGB2A2 SDF2L1 SLC30A7 SLC31A1 SNX8 ST14 TCTN2 TMEM45A TRAPPC2L WLS WNT16 WNT4 WNT5A WNT5B WNT7A*
WNT5B	*APPBP2 KLRG2 WNT5A WNT5B*
WNT11	*C1orf54 CCNDBP1 DDX58 EFTUD2 FUCA2 KLRG2 KRTAP10-7 KRTAP10-8 KRTAP12-3 KRTAP17-1 KRTAP5-7 KRTAP5-9 LDLRAD1 MDFI MKRN2 PDGFRA RASSF10 TMED7 WNT11 WNT16*

Table S2. SNPs included in this study.

Interactome	SNPs
WNT1	rs2160525_A rs1012672_T rs11609634_T rs6488506_G rs12310020_C rs16907810_C rs7974059_G rs7966410_G rs1181332_G rs17302049_G rs10743980_T rs1114157_C rs4937_T rs170365_T rs10820879_G rs9409427_A rs1388966_C rs902921_G rs6479363_C rs10991994_C rs10991995_T rs7871554_G rs7033293_G rs7850575_G rs10512215_A rs1021442_T rs10992013_C rs1532230_C rs12337695_A rs10992027_G rs1492684_T rs10992030_G rs7851193_G rs4743847_T rs1021443_C rs10739916_T rs10820886_C rs17506543_G rs9409639_G rs7036863_C rs1388967_A rs7850263_T rs2387871_A rs10992044_G rs9409644_T rs12343922_A rs1201041_C rs12339089_G rs9409645_G rs923769_G kgp9313529_T rs10992058_G rs1135150_T rs10761129_C rs10992065_T rs10992066_T rs10992071_T rs10992072_T rs4237215_A rs9409651_T rs16907720_G rs10992075_G rs9409653_A rs3935601_A rs16907725_A rs7860482_A rs10992085_A rs7026175_C rs10992087_A rs4467997_A rs10992097_C rs3935382_T rs4378021_C rs7029814_C rs4743855_C rs17587223_A rs12002478_T rs7850118_G rs4744110_G rs10761133_C rs10512219_G rs4430151_G rs16907979_C rs2001917_A rs1892263_T rs7037255_A rs7038823_T rs12914_G rs1127379_G rs7843510_G rs6651363_A rs4736964_A rs10958671_G rs17574424_T rs747417_A rs7832767_T rs968427_G rs11900648_T rs17554448_G rs12991583_T rs4581866_A rs1461961_A rs1017771_C rs13011286_A rs11691281_G rs13430186_G rs1466400_G rs10189407_G rs13408281_G rs7572721_T rs13781_A rs7562311_G rs10784434_G rs3782497_C rs7299766_C rs17101043_C rs1566320_C rs7301320_T
WNT2	rs3736309_G rs923911_A rs2098322_C rs1076458_A rs3815500_G rs12885397_A rs3742832_C kgp6061961_C kgp12063205_T rs2572_A rs7577243_A rs13424854_G rs7589322_A rs3828334_G rs3828336_A rs2292832_T rs881029_G rs12695020_G rs2228327_T rs1126920_T rs13013933_A rs3792215_T rs3787367_G rs6119761_T rs242602_C rs242608_T rs980368_G rs12009_C rs1200160_A rs12724494_T rs12758208_A rs1080266_A rs12733626_T rs10732287_T rs10919132_G rs7538259_A rs10919147_G rs16862093_G rs1400838_A rs4656670_T rs4656671_A rs6563_G rs2229974_C rs3124596_A rs3812605_T kgp9044879_T rs3125002_G rs2229971_C rs3812609_T rs3125006_A rs4489420_G rs13290979_G rs11145770_T rs3013302_A rs699780_C rs17258579_C rs17258599_C rs1131293_G rs10187363_T rs2240542_C rs2058062_A rs13382756_A rs4258787_C rs1470414_A rs10933532_T rs3806599_A rs3796902_T rs2595110_C rs7668322_G rs28497408_C rs8142229_T rs7290471_G rs12163408_A rs8142016_T rs9628184_A rs9616936_G rs7410368_T rs2334099_A rs2273257_G rs1076780_A rs2472429_C rs11228258_A rs2840367_C rs7927922_G rs12804775_A rs17499125_T rs11211460_T rs12914_G rs1127379_G rs7843510_G rs6651363_A rs4736964_A rs10958671_G rs17574424_T rs747417_A rs7832767_T rs968427_G rs661057_C rs3862605_C rs3862606_G rs3781825_C rs645275_C rs1784934_T rs11218301_A rs4631890_A rs676759_C rs17245976_T rs12364988_C rs12285564_A rs11218322_C rs3781827_G rs2276346_T rs10502262_A rs11605969_T rs7124060_T rs3781832_T rs1699105_C rs4420280_A rs4936637_C rs3781836_A rs11218349_A rs1699103_A rs1699102_C rs1503422_T

	rs1699099_C rs726601_T rs1784933_G rs1614735_G rs10790449_C rs1133174_A rs7526959_C rs11209214_C rs9436797_A rs1033975_A rs11805150_C rs9436367_G rs11209216_T rs13375520_C rs1337406_C rs269352_A rs12726865_A rs12134024_G rs17130484_G rs2566761_C rs2435841_C rs12741473_C rs2988019_C rs14130_C rs1367452_C rs12046920_G rs12740674_C rs10158359_C rs7062_A rs7552304_A rs4655576_T rs6658452_A rs2566783_C rs11209223_G rs12405510_A rs2566784_T rs983034_T rs2052959_G rs1556157_T rs1430758_A rs1430759_A rs17482018_C rs2566759_T rs1367448_T rs1367447_A rs2566757_A rs11587907_G rs1864569_C rs12132335_A rs2566755_G rs2772304_T rs4233320_T rs12037394_G rs2566754_C rs12034358_T rs944082_A rs17130546_G rs2772300_T rs891526_C rs2116046_C rs7554551_C rs7529246_G rs10889751_C rs17130585_T rs11209233_A rs10493438_T rs10493439_G rs2915124_C rs2772280_C rs4730775_T rs2024233_G rs733153_C rs2896218_G rs4727847_G rs10487362_A rs10227271_G rs6947329_T rs2285545_C rs39312_C rs8075562_A rs781858_C rs16953649_A rs7213487_G rs781831_G rs9895032_T rs9901660_G rs747360_T rs17176181_G rs1377807_G
WNT5A	rs11126210_G rs6546473_G rs11887395_A rs4315565_G rs6723425_T rs13427290_A rs4854538_C rs12713670_T kgp1362589_G rs7591556_C rs10167468_A rs4527239_T rs10203421_C rs4077467_A rs1106518_T rs4629191_G rs6710260_G rs4241349_G rs4555379_C rs11689690_T rs4854547_C rs6710147_A rs7584948_G rs4358156_T rs12105936_A rs7561207_A rs11673753_T rs3890787_G rs4488695_G rs9989781_A rs9808255_A rs10176087_A rs13016276_G rs11126220_T rs4299364_A rs11126223_G rs10182789_G rs6732795_C rs7591558_T rs7564412_A rs11126226_T rs11680427_C rs6751798_G rs7508_C rs3810_T rs420610_A rs17515291_G rs2073574_T rs12155668_T kgp3494779_A rs11986226_G rs13263632_T rs172378_G rs11979330_A rs16840041_A rs366316_G rs411089_T rs2072660_T rs3811450_T rs476474_A rs17205511_C rs494312_T rs12824889_C rs1054611_T rs10772226_A rs11053548_G rs6756482_G rs12482177_C rs8129814_C rs2277814_A rs2850174_G rs9978314_C rs2839077_T rs1053312_A kgp4735524_T rs9254_A rs3823231_G rs345730_G rs345727_A rs345725_C rs345713_T rs17448051_G rs9363058_T rs345716_C rs3857470_A rs164296_A rs164301_T rs1492964_T rs164556_G rs16871170_T rs13200186_T rs13200697_T rs7751214_T rs168435_A rs164533_T rs164538_C rs164539_T rs17688085_A rs164540_G rs164548_A rs9354011_A rs4707796_C rs2006999_G rs351328_A rs16880204_T rs10455552_T rs564158_G rs625822_C rs3799792_G rs1147694_A rs1147695_G rs1147697_T rs1147705_T rs13097755_T rs1259293_T rs1259297_G rs1259299_C rs1402372_T rs1733302_T rs1267675_A rs13326852_T rs1259339_G rs1052015_C rs7400002_C rs11842558_C rs7399657_C rs9604573_T rs7996080_T rs12868833_A rs6602910_G rs9577922_C rs7997328_C rs6602905_G rs6088624_A kgp11005472_T kgp3633912_C kgp4233699_T rs11546155_A kgp4083359_A rs17122844_T rs8107142_A rs12461158_A rs870379_C rs45512696_T rs2305744_G rs1042328_T rs12009_C rs2565163_A rs17730989_C rs13386066_G rs9632774_A rs10954649_T rs17160911_G rs12112028_T rs10441417_A rs10250296_A rs6945850_T kgp645444_T rs2811724_C rs2811728_A rs10158824_C rs3785529_A rs2076425_T rs4984954_C rs4984705_G rs760560_T rs4984962_T rs742036_T rs2277892_A rs7202025_C rs1544800_A rs12448994_T rs17146060_A rs402605_C rs9933730_G rs3751667_A rs12595929_T rs4984738_G rs2382948_A rs11248958_G rs2160525_A rs1012672_T rs11609634_T rs6488506_G rs12310020_C rs16907810_C rs7974059_G rs7966410_G rs1181332_G rs17302049_G

	rs10743980_T rs10109061_G rs977785_A rs3804465_G rs9328375_T rs10214456_C rs9328376_T rs4246076_A rs9405309_C rs9502478_T rs9328377_C rs3789765_A rs7740529_T rs909790_C rs2294461_C rs9392815_T rs3789766_C rs6934234_G rs763405_T rs3804474_T rs7770797_G rs9504873_T rs3804476_G rs6922002_A rs17142392_C rs1076673_A rs11961398_A rs11963955_G rs9502483_A rs3804485_A rs7774142_C rs3804488_A rs3804490_G rs2001202_A rs10178695_A rs9973717_T rs713535_T rs964842_G rs2255648_G rs2450855_A rs2454427_T rs713178_G rs3773341_A rs1542848_A rs9855183_T rs2344825_A rs15997_C rs5746255_C rs10821609_T rs10821610_G kgp2676079_G rs10994294_T kgp10087292_G kgp3059613_C rs10994470_A kgp10226621_A rs2285388_T rs9530622_C rs9544422_G rs17692643_G rs7337686_G rs17693337_C rs9530625_T rs9600841_G rs4885452_A rs699780_C rs17258579_C rs17258599_C rs4889852_T rs4077719_C rs11657630_A rs7678144_C rs7677751_T rs2229307_C kgp265203_C rs7656613_C rs1547904_T rs12511976_C rs3796902_T rs2595110_C rs7668322_G rs1126973_G rs6681804_C rs6694800_T rs7513553_T rs12118846_G rs3131651_G rs3122438_G rs11207493_T rs10889147_C rs6600312_T rs1158867_C rs2069912_C rs5937_C rs2069933_T rs6123_G rs4857343_T rs8178607_T rs13062355_G rs2978974_A rs2976391_A rs1045547_G rs3190930_A rs3901172_G rs6905941_C rs2024557_C rs717851_A rs6569519_C rs2326671_C rs10484714_T rs7776358_C rs10456973_T rs9482859_G rs6912749_T rs9375548_T rs17828130_A rs4498385_G rs6926332_C rs4596504_C rs1012049_A rs6923988_C rs11966128_G rs17430298_A rs17352289_A rs9491939_C rs7748155_C rs11751266_A rs3822945_A rs9398869_A rs17055854_G rs1292626_G rs7741248_G rs6914064_C rs9491960_C rs13196432_A rs17364090_C rs11751628_A rs6923822_A rs17709552_G rs13054355_A rs10493940_C rs4908107_A rs11579723_T rs11578366_A rs10981696_T rs10817464_C rs10981699_T rs6477998_A rs7851623_T rs10817465_C rs10124895_A rs4721527_A rs7456643_C rs4549678_T rs12699820_T rs10249052_T rs4442022_C rs2398666_T rs2895183_C rs7801817_T rs7125179_G rs620263_G rs1545714_C rs478745_C rs577740_G rs597576_A rs17667603_A rs476106_C rs2324002_C rs595073_C rs11221995_T rs672442_A rs530351_A rs7944700_A rs2124059_G rs704625_G rs7137946_C rs7973155_G rs11920006_C rs12639465_A rs7429392_G rs6441308_C rs6777810_T rs7647499_C rs4928066_C rs9864288_T rs7429915_T rs3826061_G rs7526959_C rs11209214_C rs9436797_A rs1033975_A rs11805150_C rs9436367_G rs11209216_T rs13375520_C rs1337406_C rs269352_A rs12726865_A rs12134024_G rs17130484_G rs2566761_C rs2435841_C rs12741473_C rs2988019_C rs14130_C rs1367452_C rs12046920_G rs12740674_C rs10158359_C rs7062_A rs7552304_A rs4655576_T rs6658452_A rs2566783_C rs11209223_G rs12405510_A rs2566784_T rs983034_T rs2052959_G rs1556157_T rs1430758_A rs1430759_A rs17482018_C rs2566759_T rs1367448_T rs1367447_A rs2566757_A rs11587907_G rs1864569_C rs12132335_A rs2566755_G rs2772304_T rs4233320_T rs12037394_G rs2566754_C rs12034358_T rs944082_A rs17130546_G rs2772300_T rs891526_C rs2116046_C rs7554551_C rs7529246_G rs10889751_C rs17130585_T rs11209233_A rs10493438_T rs10493439_G rs2915124_C rs2772280_C rs3779381_C rs2707473_A rs3801385_G rs2707466_A rs1046310_C rs10737462_T rs12131703_T rs2235529_A rs7526484_T kgp3818437_C rs1829556_G rs11918967_C rs3773608_C rs472631_C rs648872_T rs566926_A rs4765826_A rs4765827_A rs2369856_G rs11061823_A rs10848510_C rs10773958_A rs10773959_G rs11061839_G rs1029504_A rs1012093_G rs11061841_G

	rs12425453_G rs12579055_A rs7315539_A rs886537_T rs11061849_T rs10491958_T rs11061851_G rs7959613_C rs11061856_T rs16928563_C rs2107525_C rs10848523_A rs7969171_G rs11615227_A rs11830202_A rs2240506_A rs7308793_G rs4765829_C rs3825386_G rs3809268_A rs4766396_A rs1029628_G rs4766399_G rs4766400_C rs10848538_T rs11061882_C rs2270036_T rs2270037_T rs2240507_A rs2240510_T rs2240511_G rs735892_T rs735890_G rs1124479_G rs2163910_A rs1124480_C rs12487265_G rs3796316_G rs3796314_A rs873853_T rs9840696_A rs12492620_G rs934453_C rs12634816_G rs734176_A rs12634112_T rs11922919_T rs9863149_C rs12492784_A rs1433355_A rs12639607_T rs13059544_C rs13069140_A rs7641735_C rs9828013_T rs6442416_C rs9864031_G rs11128662_T
WNT5B	rs12451779_A rs17500797_C rs12162165_T rs17592831_C rs6503977_T rs9632774_A rs10954649_T rs17160911_G rs12112028_T rs10441417_A rs10250296_A rs6945850_T kgp645444_T rs1829556_G rs11918967_C rs3773608_C rs472631_C rs648872_T rs566926_A rs4765826_A rs4765827_A rs2369856_G rs11061823_A rs10848510_C rs10773958_A rs10773959_G rs11061839_G rs1029504_A rs1012093_G rs11061841_G rs12425453_G rs12579055_A rs7315539_A rs886537_T rs11061849_T rs10491958_T rs11061851_G rs7959613_C rs11061856_T rs16928563_C rs2107525_C rs10848523_A rs7969171_G rs11615227_A rs11830202_A rs2240506_A rs7308793_G rs4765829_C rs3825386_G rs3809268_A rs4766396_A rs1029628_G rs4766399_G rs4766400_C rs10848538_T rs11061882_C rs2270036_T rs2270037_T rs2240507_A rs2240510_T rs2240511_G rs735892_T rs735890_G
WNT11	rs10494266_G rs11205354_A rs10157197_A rs11584761_A rs2274127_C rs530118_C rs513071_G rs12006123_A rs10738889_G rs3205166_C rs9650702_A rs944582_G rs4384073_G rs17217280_T rs7037171_C rs10971001_G rs639949_G rs7026407_G rs11795343_C rs2231648_T rs2304987_T rs2289672_A rs9910260_A rs17628000_A rs9914884_C rs2289677_C rs8067833_T rs505481_G rs3789790_G rs11155297_T rs1038390_G rs9632774_A rs10954649_T rs17160911_G rs12112028_T rs10441417_A rs10250296_A rs6945850_T kgp645444_T rs370092_A rs411254_A kgp249076_A kgp7688819_T rs11234042_A rs2664_C rs2663_A rs10158824_C rs2495229_T rs2268411_T rs2268410_A rs16894871_G rs2842659_G rs3789208_T rs1005724_A rs4714501_A rs2230088_A rs1056684_A rs2255648_G rs2450855_A rs2454427_T rs713178_G rs3773341_A rs1542848_A rs9855183_T rs2344825_A rs15997_C rs5746255_C rs7678144_C rs7677751_T rs2229307_C kgp265203_C rs7656613_C rs1547904_T rs12511976_C kgp8004408_C rs698366_A rs11957931_T rs10075869_G rs17749202_C rs11236646_T rs7936750_G rs882151_T rs689095_G rs94111_A rs749311_A rs3779381_C rs2707473_A rs3801385_G rs2707466_A

Table S3. Single SNP interaction models identified and results of the logistic regression analyses.

a) Wnt1 interactome dataset.

µTop Model SNPs	Top Model Risk Categorization	Permutation Testing P-value	Logistic Regression P-value	Odds Ratio (OR)	Logistic Regression Confidence Interval (CI)
**1-way**					
Iteration 1					
ROR2.rs10820879_G	rs10820879_G = AA or rs10820879_G = GG → Low Risk rs10820879_G = GA → High Risk	0.012	0.004566	2.033	1.245 - 3.319
Iteration 2					
ROR2.rs1388966_C	rs1388966_C = TT → Low Risk. rs1388966_C = CT or rs1388966_C = CC → High Risk	0.143	--		--

b) Wnt2 interactome dataset.

µTop Model SNPs	Top Model Risk Categorization	Permutation Testing P-value	Logistic Regression P-value	Odds Ratio (OR)	Logistic Regression Confidence Interval (CI)
**1-way**					
Iteration 1					
HSPA5.rs12009_C	rs12009_C = TT → Low Risk rs12009_C = CT or rs12009_C = CC → High Risk	0.003	0.001297	2.654	1.464 - 4.810
Iteration 2					
PITX2.rs3796902_T	rs3796902_T = CC or rs3796902_T = TT → Low Risk	0.012	0.006400	1.941	1.205 - 3.128

	rs3796902_T = TC → High Risk				
Iteration 3					
WLS.rs7529246_G	rs7529246_G = GA → Low Risk<br>rs7529246_G = AA or<br>rs7529246_G = GG → High Risk	0.027	0.007525	2.030	1.208 - 3.413
Iteration 4					
NME7.rs1080266_A	rs1080266_A = GG or<br>rs1080266_A = AA → Low Risk<br>rs1080266_A = AG → High Risk	0.086	--	--	--

c) Wnt5a interactome dataset.

µTop Model SNPs	Top Model Risk Categorization	Permutation Testing P-value	Logistic Regression P-value	Odds Ratio (OR)	Logistic Regression Confidence Interval (CI)
**1-way**					
Iteration 1					
EPHA7.rs9363058_T	rs9363058_T = CC → Low Risk<br>rs9363058_T = TC or<br>rs9363058_T = TT → High Risk	0.005	0.006930	1.893	1.191 - 3.009
Iteration 2					
HSPA5.rs12009_C	rs12009_C = TT → Low Risk<br>rs12009_C = CT or<br>rs12009_C = CC → High Risk	0.002	0.001297	2.654	1.464 - 4.810
Iteration 3					
FSTL1.rs1259297_G	rs1259297_G = GA → Low Risk<br>rs1259297_G = AA or<br>rs1259297_G = GG → High Risk	0.033	0.01069	1.850	1.154 - 2.967
Iteration 4					
EPHA7.rs164540_G	rs164540_G = AA or<br>rs164540_G = GG → Low Risk<br>rs164540_G = GA → High Risk	0.038	0.01043	1.818	1.151 - 2.873
Iteration 5					

ST14.rs595073_C	rs595073_C = AA or<br>rs595073_C = CC → Low Risk<br>rs595073_C = CA → High Risk	0.002	0.004031	1.952	1.237 - 3.080
		Iteration 6			
HPN.rs870379_C	rs870379_C = TT → Low Risk<br>rs870379_C = CT or<br>rs870379_C = CC → High Risk	0.021	0.01252	1.831	1.139 - 2.944
		Iteration 7			
MKRN2.rs5746255_C	rs5746255_C = CT → Low Risk<br>rs5746255_C = TT or<br>rs5746255_C = CC → High Risk	0.015	0.006115	2.026	1.223 - 3.357
		Iteration 8			
PITX2.rs3796902_T	rs3796902_T = CC or<br>rs3796902_T = TT → Low Risk<br>rs3796902_T = TC → High Risk	0.017	0.006400	1.941	1.205 - 3.128
		Iteration 9			
ST14.rs2324002_C	rs2324002_C = TT or<br>rs2324002_C = CC → Low Risk<br>rs2324002_C = CT → High Risk	0.016	0.008121	1.862	1.175 - 2.951
		Iteration 10			
WLS.rs7529246_G	rs7529246_G = GA → Low Risk<br>rs7529246_G = AA or<br>rs7529246_G = GG → High Risk	0.006	0.007525	2.030	1.208 - 3.413
		Iteration 11			
EPHA7.rs564158_G	rs564158_G = GA → Low Risk<br>rs564158_G = AA or<br>rs564158_G = GG → High Risk	0.042	0.01619	1.889	1.125 - 3.173
		Iteration 12			
ST14.rs672442_A	rs672442_A = GG or<br>rs672442_A = AA → Low Risk<br>rs672442_A = AG → High Risk	0.007	0.008915	1.861	1.168 - 2.965
		Iteration 13			
HPN.rs12461158_A	rs12461158_A = GG → Low Risk<br>rs12461158_A = AG or<br>rs12461158_A = AA → High Risk	0.018	0.03278	1.642	1.041 - 2.589
		Iteration 14			

ANTXR1.rs4629191_G	rs4629191_G = AA → Low Risk<br>rs4629191_G = GA or<br>rs4629191_G = GG → High Risk	0.077	--	--	--

d) Wnt5b interactome dataset.

µTop Model SNPs	Top Model Risk Categorization	Permutation Testing P-value	Logistic Regression P-value	Odds Ratio (OR)	Logistic Regression Confidence Interval (CI)
**1-way**					
Iteration 1					
WNT5B.rs10848510_C	rs10848510_C = TT or<br>rs10848510_C = CC → Low Risk<br>rs10848510_C = CT → High Risk	0.022	0.007245	1.986	1.204 - 3.278
Iteration 2					
WNT5A.rs11918967_C	rs11918967_C = CG → Low Risk<br>rs11918967_C = GG or<br>rs11918967_C = CC → High Risk	0.022	0.03134	1.650	1.046 - 2.605
Iteration 3					
WNT5B.rs11061823_A	rs11061823_A = CC or<br>rs11061823_A = AA → Low Risk<br>rs11061823_A = AC → High Risk	0.01	0.009285	2.040	1.192 - 3.490
Iteration 4					
WNT5B.rs10773958_A	rs10773958_A = GG → Low Risk<br>rs10773958_A = AG or<br>rs10773958_A = AA → High Risk	0.075	--	--	--

e) Wnt11 interactome dataset.

µTop Model SNPs	Top Model Risk Categorization	Permutation Testing P-value	Logistic Regression P-value	Odds Ratio (OR)	Logistic Regression Confidence

					Interval (CI)
**1-way**					
Iteration 1					
MKRN2.rs5746255_C	rs5746255_C = CT → Low Risk<br>rs5746255_C = TT or<br>rs5746255_C = CC → High Risk	0.006	0.006115	2.026	1.223 - 3.357
Iteration 2					
DDX58.rs944582_G	rs944582_G = GA → Low Risk<br>rs944582_G = AA or<br>rs944582_G = GG → High Risk	0.039	0.01914	1.768	1.098 - 2.847
Iteration 3					
DDX58.rs4384073_G	rs4384073_G = GA or<br>rs4384073_G = GG → Low Risk<br>rs4384073_G = AA → High Risk	0.009	0.01101	2.098	1.185 - 3.713
Iteration 4					
MDFI.rs4714501_A	rs4714501_A = AG or<br>rs4714501_A = AA → Low Risk<br>rs4714501_A = GG → High Risk	0.207	--	--	--

High risk genotypes are indicated in red font. µThe letter at the end is the minor allele.

Table S4. SNP functional annotations (based on the dbSNP database).

Analysis	Gene	SNP	dbSNP molecular consequences
Wnt1 1-way iteration 1	ROR2	rs10820879	[no annotation]
Wnt1 2-way	ROR2	rs7037255	intron variant
	SFRP1	rs7843510	intron variant
Wnt1 3-way	LRP6	rs11609634	intron variant
	ROR2	rs7037255	intron variant
	UBR3	rs11691281	intron variant
Wnt2 1-way iteration 1	HSPA5	rs12009	3 prime UTR variant
Wnt2 1-way iteration 2	PITX2	rs3796902	intron variant
Wnt2 1-way iteration 3	WLS	rs7529246	nc transcript variant, intron variant
Wnt2 2-way	GPC1	rs12695020	intron variant
	WLS	rs2116046	intron variant
Wnt2 3-way	HCK	rs980368	intron variant
	PPP6R3	rs2840367	intron variant
	SORL1	rs3862606	intron variant
Wnt5a 1-way iteration 1	EPHA7	rs9363058	intron variant
Wnt5a 1-way iteration 2	HSPA5	rs12009	3 prime UTR variant
Wnt5a 1-way iteration 3	FSTL1	rs1259297	intron variant
Wnt5a 1-way iteration 4	EPHA7	rs164540	intron variant
Wnt5a 1-way iteration 5	ST14	rs595073	intron variant

Wnt5a 1-way iteration 6	HPN	rs870379	intron variant
Wnt5a 1-way iteration 7	MKRN2	rs5746255	3 prime UTR variant
Wnt5a 1-way iteration 8	PITX2	rs3796902	intron variant
Wnt5a 1-way iteration 9	ST14	rs2324002	intron variant
Wnt5a 1-way iteration 10	WLS	rs7529246	nc transcript variant, intron variant
Wnt5a 1-way iteration 11	EPHA7	rs564158	intron variant
Wnt5a 1-way iteration 12	ST14	rs672442	intron variant
Wnt5a 1-way iteration 13	HPN	rs12461158	intron variant
Wnt5a 2-way	FSTL1	rs1402372	intron variant
	ST14	rs704625	3 prime UTR variant
Wnt5a 3-way	LRP6	rs10743980	intron variant
	WLS	rs2915124	intron variant
	WNT5B	rs10848523	intron variant
Wnt5b 1-way iteration 1	WNT5B	rs10848510	intron variant
Wnt5b 1-way iteration 2	WNT5A	rs11918967	intron variant
Wnt5b 1-way iteration 3	WNT5B	rs11061823	intron variant
Wnt5b 2-way	WNT5B	rs10773958	intron variant
	WNT5B	rs10491958	nc transcript variant, 3 prime UTR variant, intron variant
Wnt5b 3-way	KLRG2	rs9632774	3 prime UTR variant, intron variant
	WNT5B	rs11061856	nc transcript variant, 3 prime UTR variant, intron variant

	WNT5B	rs4766399	intron variant
Wnt11 1-way iteration 1	MKRN2	rs5746255	3 prime UTR variant
Wnt11 1-way iteration 2	DDX58	rs944582	intron variant
Wnt11 1-way iteration 3	DDX58	rs4384073	intron variant
Wnt11 2-way	FUCA2	rs11155297	missense variant
	TMED7	rs10075869	intron variant
Wnt11 3-way	C1orf54	rs10157197	intron variant
	TMED7	rs698366	3 prime UTR variant, intron variant
	WNT11	rs17749202	3 prime UTR variant

Information retrieved from the "Molecular consequences" column in the Variation Data section of the "dbSNP [1] Variation Viewer. nc variant: non-coding variant.

Table S5. Information on the genes.

a) Genes in the interactome sets.

Analysis	Gene	*Gene function/name	**Relation to colorectal cancer
Wnt1	ROR2	Receptor tyrosine kinase like orphan receptor 2	Regulates non-canonical Wnt genes in a colon cell line[2]; cytoplasmic ROR2 expression is associated with disease stage, lymph node metastasis status, and survival times in colorectal cancer patients [3]
	SFRP1	Secreted frizzled related protein 1	Downregulated and associated with overall survival in the TCGA colon cancer cohort [4]
	LRP6	LDL receptor related protein 6	Upregulated in colorectal tumors [5]; when activated/over-expressed promoted migration of colon cancer cell lines [6]
	UBR3	Ubiquitin protein ligase E3 component n-recognin 3	n/a
Wnt2	HSPA5	Heat shock protein family A (Hsp70) member 5	Involved in colorectal cancer [7]
	PITX2	Paired like homeodomain 2	Leads to invasive features in colon cancer cells through activating Wnt pathway [8]; expression levels are associated with patient survival times [9]
	WLS	Wnt ligand secretion mediator	Expression levels are associated with patient prognosis [10]
	GPC1	Glypican 1	Expression levels are associated with patient prognosis in the TCGA cohort [11]; involved in proliferation and migration in cell lines [11]
	HCK	HCK proto-oncogene, Src family tyrosine kinase	Expression levels are associated with patient survival and local inflammatory response [12]
	PPP6R3	Protein phosphatase 6 regulatory subunit 3	n/a
	SORL1	Sortilin related receptor 1	n/a
Wnt5a	EPHA7	EPH receptor A7	n/a
	HSPA5	Heat shock protein family A (Hsp70) member 5	Involved in colorectal cancer [7]
	FSTL1	Follistatin like 1	Upregulated in colorectal tumors and associated with metastasis and prognosis in colorectal cancer patients [13]

	ST14	ST14 transmembrane serine protease matriptase	Tumor suppressive function in mouse colon [14]
	HPN	Hepsin	Expression levels are associated with risk of metastasis and prognosis in colorectal cancer patients [15]
	MKRN2	Makorin ring finger protein 2	Deletion of this gene is associated with prognosis in TCGA colorectal cancer dataset [16]
	PITX2	Paired like homeodomain 2	Leads to invasive features in colon cancer cells through activating Wnt pathway [8]; expression levels are associated with patient survival times [9]
	WLS	Wnt ligand secretion mediator	Expression levels are associated with patient prognosis [10]
	LRP6	LDL receptor related protein 6	Upregulated in colorectal tumors [5]; when activated/over-expressed promoted migration of colon cancer cell lines [6]
	WNT5B	Wnt family member 5B	Oncogenic role in colorectal cancer [17]
Wnt5b	WNT5B	Wnt family member 5B	Oncogenic role in colorectal cancer [17]
	WNT5A	Wnt family member 5A	Oncogenic and tumor suppressor effects in colorectal cancer [17,18]
	KLRG2	Killer cell lectin like receptor G2	n/a
Wnt11	MKRN2	Makorin ring finger protein 2	Deletion of this gene is associated with prognosis in TCGA colorectal cancer dataset [16]
	DDX58	RNA sensor RIG-I	Important in preventing proliferation, migration and invasion of human colon cancer cells [19]
	FUCA2	Alpha-L-fucosidase 2	n/a
	TMED7	Transmembrane p24 trafficking protein 7	n/a
	C1orf54	Chromosome 1 open reading frame 54	n/a
	WNT11	Wnt family member 11	Oncogenic roles in colorectal cancer [17]

b) Genes associated with eQTLs.

1-WAY						
**µVariant**	**GTEx eQTL in colon sigmoid - target gene**	***Gene function/name**	****Relation to colorectal cancer**	**GTEx eQTL in colon transverse - target gene**	***Gene function/name**	****Relation to colorectal cancer**
HSPA5.rs12009_C	PRPS1P2	Phosphoribosyl pyrophosphate synthetase 1 pseudogene 2	n/a	PRPS1P2	Phosphoribosyl pyrophosphate synthetase 1 pseudogene 2	n/a
MKRN2.rs5746255_C	MKRN2	Makorin ring finger protein 2	Deletion of this gene is associated with prognosis in TCGA colorectal cancer dataset [16]	MKRN2	Makorin ring finger protein 2	Deletion of this gene is associated with prognosis in TCGA colorectal cancer dataset [16]
DDX58.rs944582_G	GVQW1; ACO1	n/a; Aconitase 1	n/a; Tumor expression levels are associated with prognosis in rectal but not colon cancer in the TCGA cohort [20]	DDX58; ACO1	RNA sensor RIG-I; aconitase 1	Important in preventing proliferation, migration and invasion of human colon cancer cells [19]; tumor expression levels are associated with prognosis in rectal but not colon cancer in the TCGA cohort [20]
DDX58.rs4384073_G	ACO1	Aconitase 1	Tumor expression levels are associated with prognosis in rectal but not colon cancer in the TCGA cohort [20]	No		

NME7.rs1080266_A	NME7	NME/NM23 family member 7	n/a	NME7	NME/NM23 family member 7	n/a
**2-WAY**						
ROR2.rs7037255_A	No			SPTLC1	Serine palmitoyltransferase long chain base subunit 1	n/a
SFRP1.rs7843510_G	No			No	-	
GPC1.rs12695020_G	n/a	-	-	n/a	-	-
WLS.rs2116046_C	No			GNG12-AS1	GNG12, DIRAS3 and WLS antisense RNA 1	n/a
FUCA2.rs11155297_T	ADAT2	Adenosine deaminase tRNA specific 2	n/a	ADAT2	Adenosine deaminase tRNA specific 2	n/a
TMED7.rs10075869_G	AC010226.4	n/a		AC010226.4	n/a	n/a
**3-WAY**						
LRP6.rs11609634 _T	No	-	-	No	-	-
ROR2.rs7037255_A	No	-	-	SPTLC1	Serine palmitoyltransferase long chain base subunit 1	n/a
UBR3.rs11691281_G	No	-	-	No	-	
HCK.rs980368_G	No	-	-	RP11-358N2.2	n/a	n/a
PPP6R3.rs2840367_C	No	-	-	No	-	-
SORL1.rs3862606_G	No	-	-	No	-	-

C1orf54.rs10157197_A	MRPS21	Mitochondrial ribosomal protein S21		MRPS21	Mitochondrial ribosomal protein S21	
TMED7.rs698366_A	AC010226.4; TICAM2	n/a; TIR domain containing adaptor molecule 2	n/a: Upregulated in MSI colon tumors [21]	AC010226.4; TICAM2	n/a; TIR domain containing adaptor molecule 2	n/a; Upregulated in MSI colon tumors [21]
WNT11.rs17749202_C	No	-		No	-	

*Based on the information in Gene Entrez [22] (accessed on November 7, 2022). **PUBMED was searched with the gene symbol together with "colorectal cancer" (accessed on November 7, 2022). According to RegulomeDB [23], two of these SNPs (HSPA5.rs12009_C and MKRN2.rs5746255_C) are eQTLs for *RABEPK* (Rab9 effector protein with kelch motifs), and *PPARG* (peroxisome proliferator activated receptor gamma) and *MKRN2* (makorin ring finger protein 2), respectively, in monocytes. Therefore, here we prioritized and focused on the data retrieved from the GTEx database [24]. n/a: not available. µThe letter at the end is the minor allele. MSI: microsatellite instable.

Figure S1. Kaplan Meier curves for select 2-way and 3-way interactions.

a. Wnt1 2-way

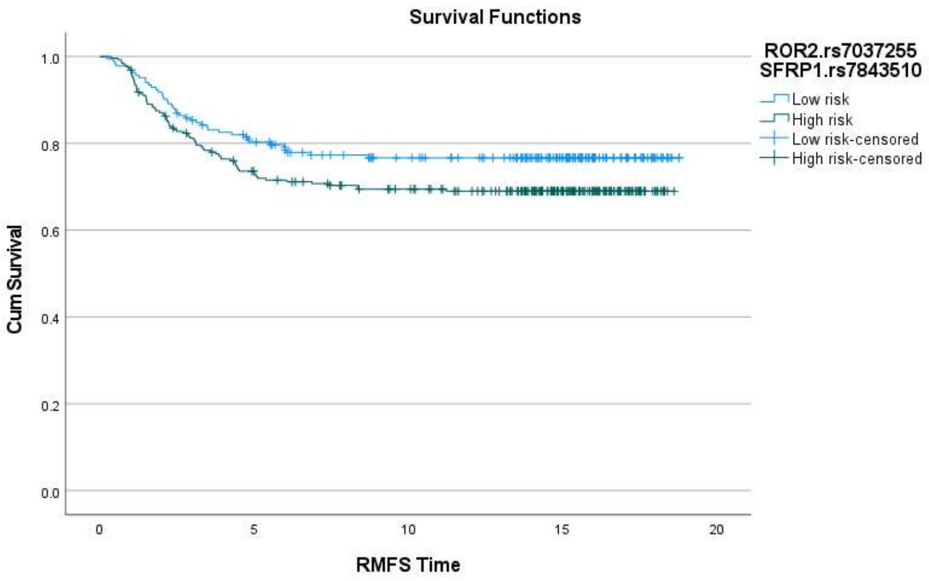

Log rank p-value: 0.07795

b. Wnt1 3-way

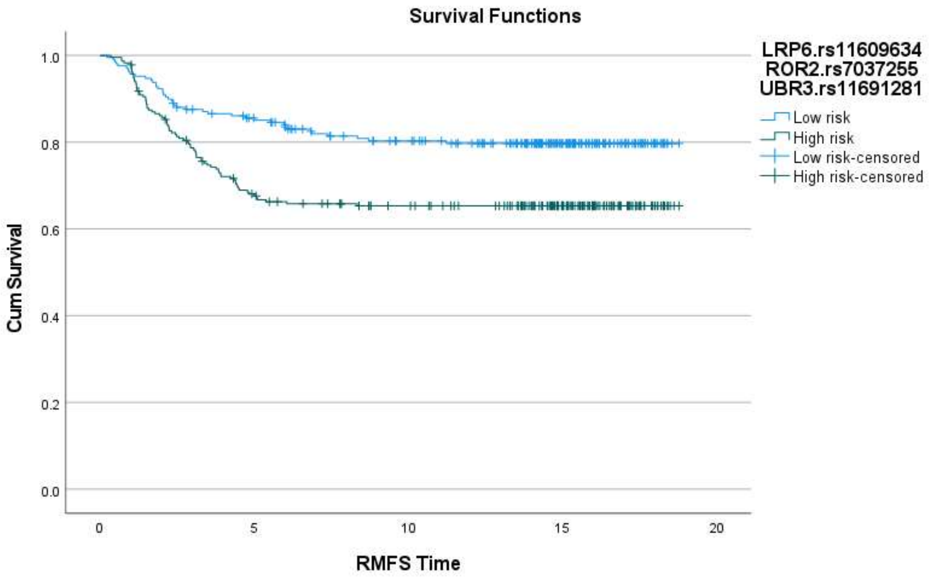

Log rank p-value: 5.816×10^{-4}

c. Wnt2 -2-way

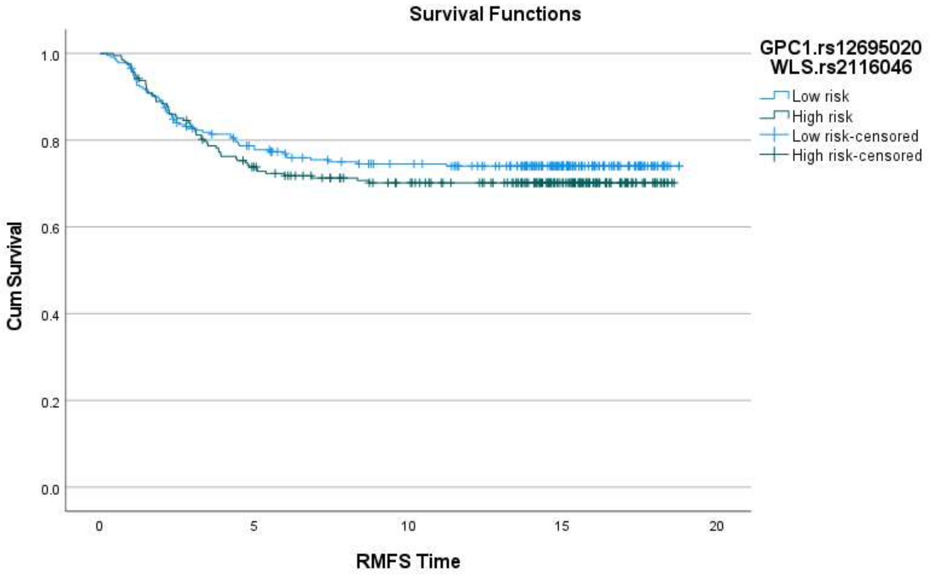

Log rank p-value: 0.4045

d. Wnt2 3-way

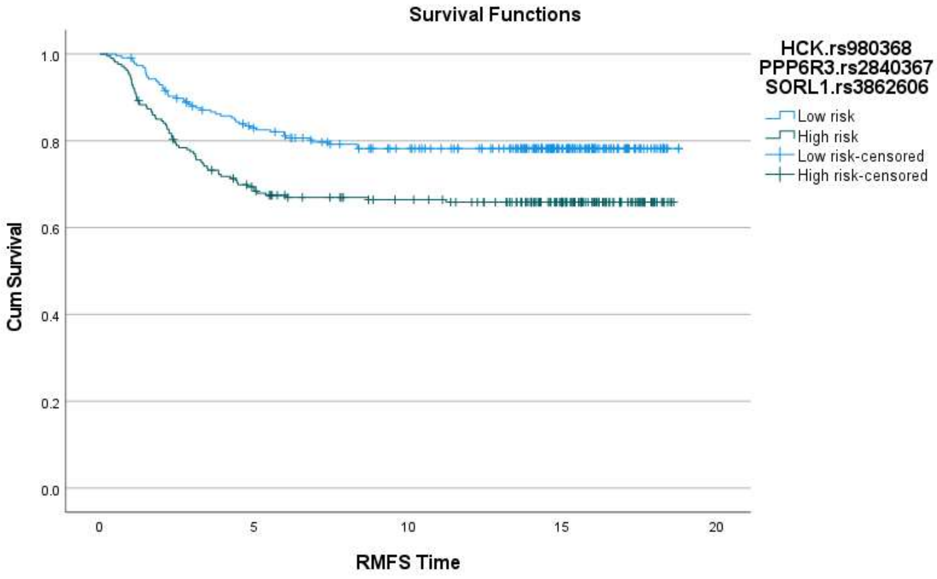

Log rank p-value: 0.001844

e. Wnt5b 3-way

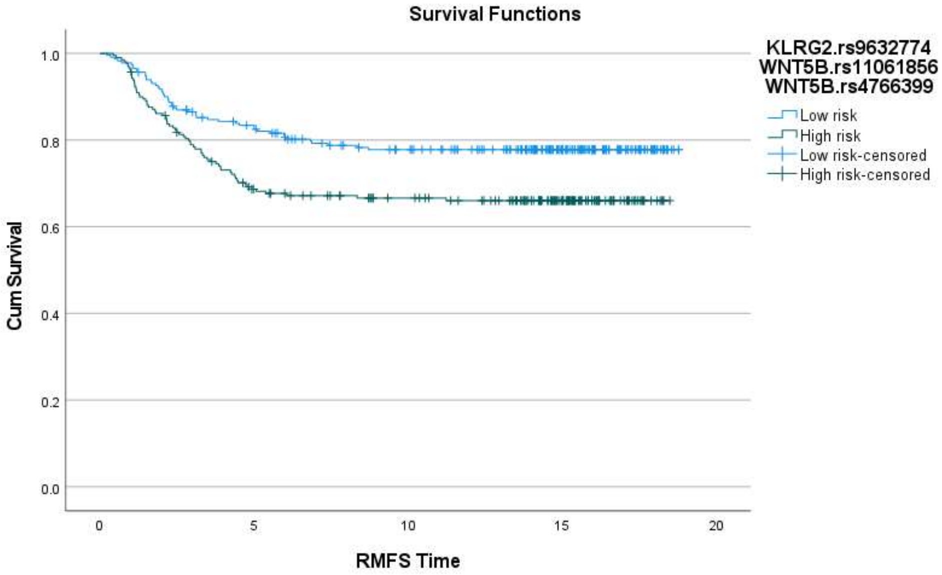

Log rank p-value: 0.004789

f. Wnt11 2-way

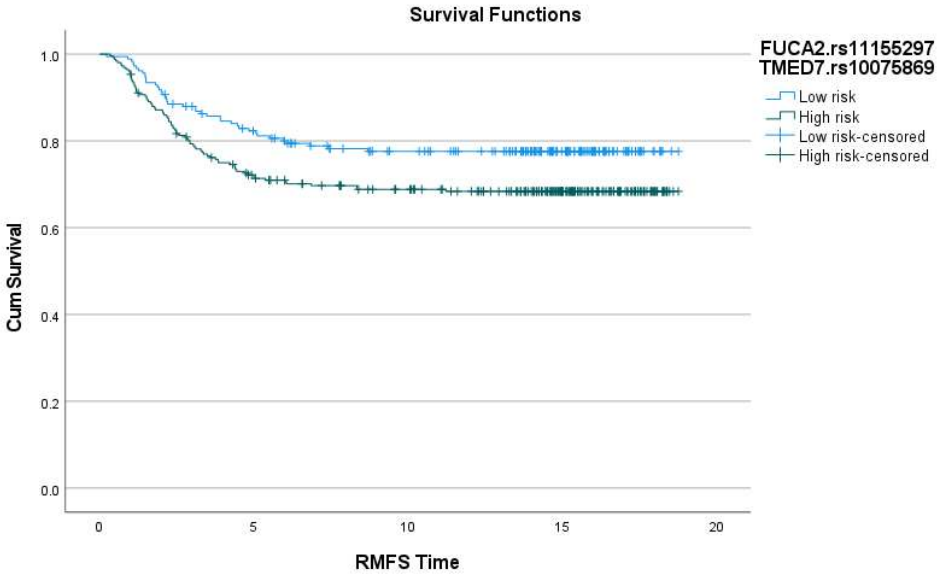

Log rank p-value: 0.02705

g. Wnt11 3-way

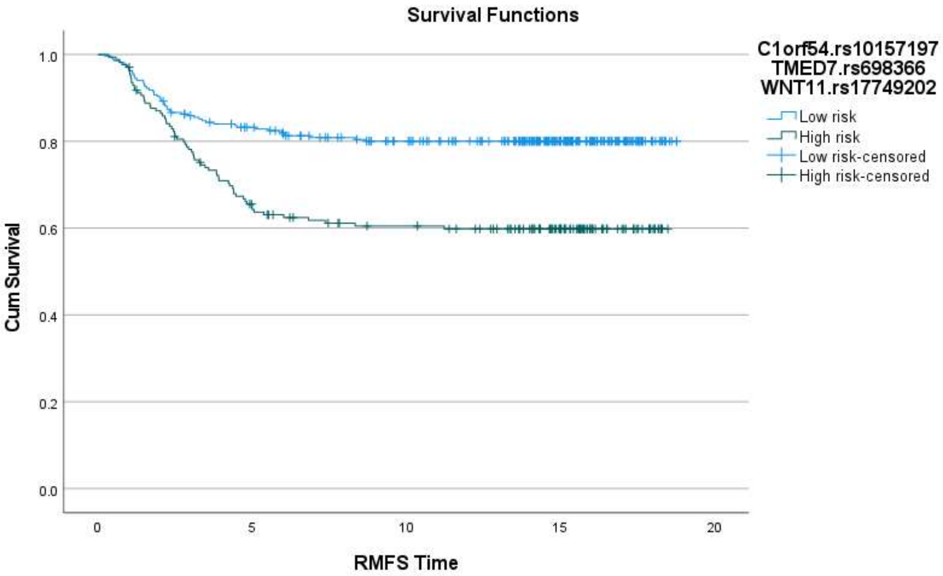

Log rank p-value: $1.068x10^{-5}$

RMFS: recurrence and metastasis-free survival. The RMFS time is shown in years.

References

1. Sherry ST, Ward MH, Kholodov M, et al. dbSNP: the NCBI database of genetic variation. *Nucleic Acids Res*. 2001;29(1):308-311.

2. Voloshanenko O, Schwartz U, Kranz D, et al. β-catenin-independent regulation of Wnt target genes by RoR2 and ATF2/ATF4 in colon cancer cells. *Sci Rep*. 2018;8:3178. doi:10.1038/s41598-018-20641-5.

3. Mei H, Lian S, Zhang S, Wang W, Mao Q, Wang H. High expression of ROR2 in cancer cell correlates with unfavorable prognosis in colorectal cancer. *Biochemical and Biophysical Research Communications*. 2014;453(4):703-709. doi:10.1016/j.bbrc.2014.09.141.

4. Busuioc C, Ciocan-Cartita CA, Braicu C, et al. Epithelial-mesenchymal transition gene signature related to prognostic in colon adenocarcinoma. *J Pers Med*. 2021;11(6):476. doi:10.3390/jpm11060476.

5. Rismani E, Fazeli MS, Mahmoodzadeh H, et al. Pattern of LRP6 gene expression in tumoral tissues of colorectal cancer. *Cancer Biomarkers*. 2017;19(2):151-159. doi:10.3233/CBM-160175.

6. Yao Q, An Y, Hou W, et al. LRP6 promotes invasion and metastasis of colorectal cancer through cytoskeleton dynamics. *Oncotarget*. 2017;8(65):109632-109645. doi:10.18632/oncotarget.22759.

7. Wang R, Hua L, Ma P, et al. HSPA5 repressed ferroptosis to promote colorectal cancer development by maintaining GPX4 stability. *Neoplasma*. 2022;69(5):1054-1069. doi:10.4149/neo_2022_220331N363.

8. He Y, Gong P, Wang S, Xu Q, Chen J. The significance of homeodomain transcription factor 2 in colon cancer cells. *BioMedical Engineering OnLine*. 2021;20(1):81. doi:10.1186/s12938-021-00912-5.

9. Hirose H, Ishii H, Mimori K, et al. The Significance of PITX2 overexpression in human colorectal cancer. *Ann Surg Oncol*. 2011;18(10):3005-3012. doi:10.1245/s10434-011-1653-z.

10. Xu H, Jiang W, Zhu F, Zhu C, Wei J, Wang J. Expression of Wntless in colorectal carcinomas is associated with invasion, metastasis, and poor survival. *APMIS*. 2016;124(6):522-528. doi:10.1111/apm.12534.

11. Lu F, Chen S, Shi W, Su X, Wu H, Liu M. GPC1 promotes the growth and migration of colorectal cancer cells through regulating the TGF-β1/SMAD2 signaling pathway. *PLoS One*. 2022;17(6):e0269094. doi:10.1371/journal.pone.0269094.

12. Roseweir AK, Powell AGMT, Horstman SL, et al. Src family kinases, HCK and FGR, associate with local inflammation and tumour progression in colorectal cancer. *Cell Signal*. 2019;56:15-22. doi:10.1016/j.cellsig.2019.01.007.

13. Gu C, Wang X, Long T, et al. FSTL1 interacts with VIM and promotes colorectal cancer metastasis via activating the focal adhesion signalling pathway. *Cell Death Dis*. 2018;9(6):1-14. doi:10.1038/s41419-018-0695-6.

14. Kosa P, Szabo R, Molinolo AA, Bugge TH. Suppression of Tumorigenicity-14, encoding matriptase, is a critical suppressor of colitis and colitis-associated colon carcinogenesis. *Oncogene*. 2012;31(32):3679-3695. doi:10.1038/onc.2011.545.

15. Zaragoza-Huesca D, Nieto-Olivares A, García-Molina F, et al. Implication of Hepsin from primary tumor in the prognosis of colorectal cancer patients. *Cancers*. 2022;14(13):3106. doi:10.3390/cancers14133106.

16. Chang Z, Liu X, Zhao W, Xu Y. Identification and characterization of the copy number dosage-sensitive genes in colorectal cancer. *Molecular Therapy - Methods & Clinical Development*. 2020;18:501-510. doi:10.1016/j.omtm.2020.06.020.

17. Nie X, Liu H, Liu L, Wang YD, Chen WD. Emerging roles of Wnt ligands in human colorectal cancer. *Front Oncol*. 2020;10:1341. doi:10.3389/fonc.2020.01341.

18. Pashirzad M, Sathyapalan T, Sahebkar A. Clinical importance of Wnt5a in the pathogenesis of colorectal cancer. Pandurangan A, ed. *Journal of Oncology*. 2021;2021:1-8. doi:10.1155/2021/3136508.

19. Deng Y, Fu H, Han X, et al. Activation of DDX58/RIG-I suppresses the growth of tumor cells by inhibiting STAT3/CSE signaling in colon cancer. *International Journal of Oncology*. 2022;61(4):1-13. doi:10.3892/ijo.2022.5410.

20. Cui Y, Han B, Zhang H, Liu H, Zhang F, Niu R. Identification of metabolic-associated genes for the prediction of colon and rectal adenocarcinoma. *OTT*. 2021;Volume 14:2259-2277. doi:10.2147/OTT.S297134.

21. Slattery ML, Mullany LE, Sakoda L, et al. The NF-κB signalling pathway in colorectal cancer: associations between dysregulated gene and miRNA expression. *J Cancer Res Clin Oncol*. 2018;144(2):269-283. doi:10.1007/s00432-017-2548-6.

22. Maglott D, Ostell J, Pruitt KD, Tatusova T. Entrez Gene: gene-centered information at NCBI. *Nucleic Acids Res*. 2011;39(Database issue):D52-7. doi:10.1093/nar/gkq1237.

23. Boyle AP, Hong EL, Hariharan M, et al. Annotation of functional variation in personal genomes using RegulomeDB. *Genome Res*. 2012;22(9):1790-1797. doi:10.1101/gr.137323.112;

24. Lonsdale J, Thomas J, Salvatore M, et al. The Genotype-Tissue Expression (GTEx) project. *Nat Genet*. 2013;45(6):580-585. doi:10.1038/ng.2653.
